# Statin Therapy Is Associated with Improved Survival in Patients with Non-Serous-Papillary Epithelial Ovarian Cancer: A Retrospective Cohort Analysis

**DOI:** 10.1371/journal.pone.0104521

**Published:** 2014-08-13

**Authors:** Mohammed Habis, Kristen Wroblewski, Michael Bradaric, Nadia Ismail, S. Diane Yamada, Lacey Litchfield, Ernst Lengyel, Iris L. Romero

**Affiliations:** 1 Department of Obstetrics & Gynecology/Section of Gynecologic Oncology – Center for Integrative Science, University of Chicago, Chicago, Illinois, United States of America; 2 Department of Health Studies, University of Chicago, Chicago, Illinois, United States of America; 3 Department of Pharmaceutical Sciences, Chicago State University, Chicago, Illinois, United States of America; Baylor College of Medicine, United States of America

## Abstract

**Aim:**

To determine whether statin use is associated with improved epithelial ovarian cancer (OvCa) survival.

**Methods:**

This is a single-institution retrospective cohort review of patients treated for OvCa between 1992 and 2013. Inclusion criteria were International Federation of Gynecology and Obstetrics (FIGO) stage I–IV OvCa. The primary exposures analyzed were hyperlipidemia and statin use. The primary outcomes were progression-free survival (PFS) and disease-specific survival (DSS).

**Results:**

442 patients met inclusion criteria. The cohort was divided into three groups: patients with hyperlipidemia who used statins (n = 68), patients with hyperlipidemia who did not use statins (n = 28), and patients without hyperlipidemia (n = 346). OvCa outcomes were evaluated. When we analyzed the entire cohort, we found no significant differences in PFS or DSS among the groups. The median PFS for hyperlipidemics using statins, hyperlipidemics not using statins, and non-hyperlipidemics was 21.7, 13.6, and 14.7 months, respectively (p = 0.69). Median DSS for hyperlipidemics using statins, hyperlipidemics not using statins, and non-hyperlipidemics was 44.2, 75.7, and 41.5 months, respectively (p = 0.43). These findings did not change after controlling for confounders. However, a secondary analysis revealed that, among patients with non-serous-papillary subtypes of OvCa, statin use was associated with a decrease in hazards of both disease recurrence (adjusted HR = 0.23, p = 0.02) and disease-specific death (adjusted HR = 0.23, p = 0.04). To augment the findings in the retrospective cohort, the histology-specific effects of statins were also evaluated *in vitro* using proliferation assays. Here, statin treatment of cell lines resulted in a variable level of cytotoxicity.

**Conclusion:**

Statin use among patients with non-serous-papillary OvCa was associated with improvement in both PFS and DSS.

## Introduction

Cardiovascular disease is the leading cause of death of both men and women in the United States [1]. One of the primary means to prevent cardiovascular disease is to lower serum low-density lipoprotein (LDL) cholesterol levels using statin drugs (statins) [2], [3]. In November 2013, the American College of Cardiology/American Heart Association released guidelines recommending statin therapy for all individuals with LDL>190 mg/dL and individuals age 40–75 years with type II diabetes or a 10-year risk of CVD>7.5% [4]. Due to these new guidelines, up to 31% of Americans aged 40–75 without CVD may eventually be using statins [5].

Interestingly, statins may safeguard against cancer, as well as provide cardio-protective effects. In epidemiologic studies, a decrease in cancer incidence has been reported among statin users, with hazard ratios (HR) of cancer for statin users compared to individuals not taking a statin ranging from 0.75 to 0.80 [6], [7]. Statin use is also associated with improved cancer survival. A large study of the Danish population reported that statin users had a HR of cancer death of 0.80 (95% CI, 0.83–0.87) compared to patients who had never used statins [8]. With site-specific cancers, statin use is associated with decreased incidence and increased survival in colorectal [9]–[11], breast [12], [13] and prostate [14], [15] cancers.

A protective effect of statins in cancer is biologically plausible. Statins lower cholesterol by inhibiting HMG-CoA reductase, which catalyzes the first and rate-limiting step in cholesterol biosynthesis, the conversion of HMG-CoA to mevalonic acid [16]. Decreased intracellular cholesterol causes an increase in LDL receptor expression and a consequent decrease in serum LDL levels, both of which provide the cardio-protective effects of statins. This reduction in intracellular cholesterol may also convey anti-cancer effects, since rapidly dividing cancer cells require cholesterol for synthesis of cell membranes [17], [18]. A second mechanism through which statins may interfere with carcinogenesis involves reduced synthesis of the mevalonic acid pathway intermediates, isoprenoids [19]. Isoprenoids are important in the prenylation and activation of several small GTP-ase cancer signaling pathways, including Ras, Rac and Rho [20]. *In vitro* studies have shown that the inhibition of isoprenoids is one of the mechanisms mediating the effect of statins in cancer [21]–[23].

If commonly used medications, like statins, are found to have anti-cancer effects, it would be particularly relevant for diseases such as ovarian cancer (OvCa), which has a very poor prognosis. Despite years of research, there have been few new effective OvCa treatments and up to 76% of patients have recurrence of disease after first line therapy [24]. However, there is little data on the effects of statins in OvCa. In 2008, Elmore *et al.* analyzed 128 patients and found that median progression-free survival for statin users (n = 17) was 24 months, compared to 16 months for statin non-users, and that median overall survival for statin users was 62 months, compared to 46 months for statin non-users [25]. A small number of preclinical studies have also suggested a protective effect of statins in OvCa. Specifically, there have been two reports indicating that statins induce apoptosis in OvCa cells [26], [27] and one report of decreased spread of cancer in a mouse model treated with statins [22]. While these initial reports are promising, it remains unknown whether use of statins improves OvCa survival.

Given the increasing epidemiologic evidence of a protective effect of statins in various cancers and a sound biological mechanism supported by published preclinical data, we hypothesized that, in patients with OvCa, use of statins would be associated with increased cancer survival. To test this hypothesis, we analyzed a single institution retrospective cohort of OvCa patients, and compared survival between patients who used statins and those who did not use statins.

## Materials and Methods

### Patient Database

This is a retrospective cohort study of patients consecutively treated for epithelial OvCa at the University of Chicago between 1992 and 2013. All women who had treatment for Fédération Internationale de Gynécologie et d'Obstétrique (FIGO) stage I–IV epithelial ovarian, fallopian, or peritoneal cancer were selected for the study. Exclusion criteria included noninvasive pathology or non-epithelial malignancies. Gynecologic oncologists cared for all the patients and a sub-specialty trained gynecologic pathologist confirmed all pathologic diagnoses. For each patient, OvCa clinico-pathologic parameters, treatment, and outcomes were recorded and stored in a Microsoft Access database as previously reported [28], [29]. Clinical information in the database was updated every three months through February 2013. Follow-up data was obtained from the patients' charts at the University of Chicago hospital and outpatient clinic, the Illinois Cancer Registry, the United States Social Security Index, and by communicating with the physicians involved in the patient's care. The race recorded was the self-reported race listed in the patient's medical record. A data manager and an attending gynecologic oncologist (EL) reviewed all patient information. In addition to the OvCa variables, data concerning diagnoses of hyperlipidemia and hyperlipidemia medications was abstracted (MH) from outpatient and inpatient records. The person abstracting this data was unaware of the subject's OvCa survival status and one third of the records were re-reviewed by a second blinded investigator (IR).

### Ethics Statement

The Institutional Review Board at the University of Chicago (IRB #13248A) approved the study. Written informed consent was obtained from patients allowing the use of data from their clinical records to analyze OvCa survival. This study did not use any unpublished *de novo* cell lines. All cell lines reported here have a previously published reference included.

### Statistical Analysis

The primary exposures of interest for this study were a history of hyperlipidemia and use of a statin. To avoid a potential immortal time bias [30], [31], statin use was defined as taking a statin at the time of, or up to one year after, the diagnosis of OvCa, and continued use at the time of death or end of the study. The outcome measures included both progression-free survival (PFS) and disease-specific survival (DSS). Recurrence was defined using previously published clinical criteria [32] and included any evidence of the reappearance of cancer by clinical exam (e.g. tumor, ascites), new tumor findings on CT scan or ultrasound, or an increase in CA-125 greater than or equal to two times the upper limit of normal. PFS was calculated from the date of the start of treatment to the date of recurrence or death. Patients without recurrence or death were censored at last follow-up. DSS was calculated from the date of the start of treatment to the date of death from OvCa. Those still alive at their last follow-up were censored at that time. The 23 patients who died from causes other than OvCa or had an unknown cause of death were censored at the time of death.

All statistical analyses were performed using Stata version 13 (StataCorp., College Station, TX). For comparison, the cohort was stratified into three groups: hyperlipidemics using statins, hyperlipidemics not using statins, and non-hyperlipidemics. Demographic and clinico-pathologic variables were compared among the groups using analysis of variance (ANOVA) for continuous variables and Fisher's exact test for categorical variables. Kaplan-Meier survival curves were plotted for the three groups and compared with log-rank tests. A Cox proportional hazards model was used to estimate hazard ratios for PFS and DSS while adjusting for potential confounders such as age, race, BMI, smoking status, comorbidities, ASA class, surgery characteristics, histologic subtype, FIGO stage, tumor site and grade. Metformin use was also adjusted for in the models, since it has been associated with improved OvCa survival [33], [34]. Ninety-five percent confidence intervals (95% CIs) for the HR were calculated. For model selection, univariate Cox regression was run with each of the potential confounders and those found to be significant in predicting PFS or DSS were included in the final model. For the Cox regression models comparing the three groups, patients without hyperlipidemia were the reference group. We also examined hyperlipidemics using statins relative to hyperlipidemics not using statins. Statistical significance was defined as *p*<0.05.

### *In Vitro* Proliferation Assays

Lovastatin was chemically modified from the pro-drug to the active form of the drug as previously described [35]. Briefly, 50 mg of lovastatin was solubilized in 970 µL of 100% ethanol, diluted with 782 µL of 1 M NaOH and incubated at 50°C for 30 minutes. The solution was brought up to a final volume of 13 mL to make a 10 mM stock solution.

The effects of lovastatin on cancer cell proliferation were tested using MTT (3-(4,5-dimethylthiazol-2-yl)-2,5-diphenyltetrazolium bromide) proliferation assays. OvCa cells were treated with varying concentrations of lovastatin (10 µM, 20 µM and 40 µM) for 24 hours, and the rate of cellular proliferation was measured as previously described [36]. Lovastatin was purchased from Cayman Chemical (Ann Arbor, MI). The cell lines used included: SKOV3ip1 [37] (obtained from Gordon B. Mills at MD Anderson Cancer Center), OVCAR-5 [38] (purchased from American Type Culture Collection), ES-2 [39] (obtained from University of California-San Francisco), EG [40] (obtained from Anil Sood at MD Anderson Cancer Center), RMUG-S [41] and TYK-nu [42] (obtained from Kenjiro Sawada at Osaka University Graduate School of Medicine). The histologic subtype of the cells was assumed based on a published summary of OvCa cell lines [43] and the original publication, except for the TYK-nu cell line. Recent genetic profiling indicates a high likelihood that the TYK-nu cell line is serous-papillary subtype [44]. Cell lines were validated by short tandem repeat (STR) DNA fingerprinting using the AMPF'STR Identifier kit (Applied Biosystems) and compared with ATCC and University of Texas MD Anderson Cancer Center fingerprints.

## Results

Of the 442 women with OvCa included in the analysis, 68 (15%) had hyperlipidemia and used statins, 28 (6%) had hyperlipidemia and did not use statins, and 346 (78%) did not have hyperlipidemia. The three groups were similar with respect to race, BMI and smoking status (Table 1). On average, women with hyperlipidemia using statins were older, had worse general health at the time of OvCa diagnosis (i.e. higher American Society of Anesthesiologists (ASA) physical status scores) and had a higher prevalence of diabetes and cardiovascular disease. Since patients with worse general health may receive less aggressive treatment for OvCa, we first determined whether those with hyperlipidemia received similar treatment for OvCa as those without hyperlipidemia. OvCa treatment was similar among the groups (Table 1). Specifically, the rate of residual tumor <1 cm after cytoreductive surgery, type of chemotherapy and the number of chemotherapy cycles received were not significantly different between the three groups. The non-hyperlipidemic group was somewhat more likely to have primary cytoreductive surgery rather than neoadjuvant chemotherapy (*p* = 0.07). Evaluation of pathological characteristics revealed similar tumor stage, grade, and histologic subtypes among the groups. However, the patients with hyperlipidemia using statins were more likely to have fallopian tube as the tumor site (Table 2).

**Table 1:** Baseline characteristics of study group.

Parameter	Hyperlipidemia using Statins	Hyperlipidemia not using Statins	No hyperlipidemia	P value
**N**	68 (15%)	28 (6%)	346 (78%)	
Age at diagnosis (years)	67±11	62±10	58±12	<0.01
**Race**				0.38
White	44 (65%)	21 (75%)	246 (75%)	
African American	17 (25%)	6 (21%)	64 (20%)	
Other	7 (10%)	1 (4%)	18 (5%)	
*Not recorded*	*0*	*0*	*18*	
**BMI (kg/m^2^)**	29±6	27±6	28±7	0.54
*Not recorded*	*3*	*0*	*18*	
**Current smoker**	9 (13%)	3 (11%)	50 (15%)	0.97
*Not recorded*	*0*	*1*	*2*	
**Comorbidities**				
Diabetes	22 (32%)	1 (4%)	35 (10%)	<0.01
Metformin user	15 (22%)	1 (4%)	14 (4%)	<0.01
Cardiovascular disease	17 (25%)	2 (7%)	14 (4%)	<0.01
**ASA class >II**	47 (77%)	11 (48%)	147 (48%)	<0.01
*Not recorded*	*7*	*5*	*40*	
Surgery				
Primary cytoreductive	50 (74%)	21 (75%)	291 (84%)	0.07
Residual Tumor >1 cm	18 (27%)	9 (35%)	111 (34%)	0.56
*Not recorded*	*1*	*2*	*16*	
**Chemotherapy** *				
Platinum+Taxane	62 (97%)	27 (100%)	302 (94%)	0.46
≥6 cycles*	51 (82%)	17 (68%)	256 (82%)	0.25
*Not recorded*	*2*	*2*	*9*	

Data are n (%) or mean ± standard deviation.

*p*-values are from ANOVA for continuous variables and Fisher's exact test for categorical variables.

*Patients who did not receive chemotherapy were excluded from the analysis.

**Table 2:** Tumor characteristics of study group.

Parameter	Hyperlipidemia using Statins	Hyperlipidemia not using Statins	No hyperlipidemia	P value
**N**	68 (15%)	28 (6%)	346 (78%)	
**FIGO Stage**				0.94
I	9 (13%)	2 (7%)	46 (13%)	
II	4 (6%)	2 (7%)	20 (6%)	
III	42 (63%)	19 (68%)	203 (59%)	
IV	12 (18%)	5 (18%)	76 (22%)	
*Not recorded*	*1*	*0*	*1*	
**Grade**				0.58
I	2 (3%)	3 (11%)	24 (7%)	
II	14 (21%)	6 (21%)	64 (19%)	
III	51 (76%)	19 (68%)	251 (74%)	
*Not recorded*	*1*	*0*	*7*	
**Tumor site**				<0.01
Fallopian	22 (32%)	4 (14%)	41 (12%)	
Ovary	35 (51%)	20 (71%)	279 (81%)	
Peritoneum	11 (16%)	4 (14%)	25 (7%)	
*Not recorded*	*0*	*0*	*1*	
**Histologic Subtype**				0.83
Serous-papillary	54 (79%)	24 (86%)	256 (75%)	
Endometrioid	7 (10%)	2 (7%)	37 (11%)	
Clear cell	6 (9%)	1 (4%)	32 (9%)	
Mucinous	1 (1%)	1 (4%)	18 (5%)	
*Not recorded*	*0*	*0*	*3*	

Data are n (%).

*p*-values are from Fisher's exact tests.

**Figure 1:**
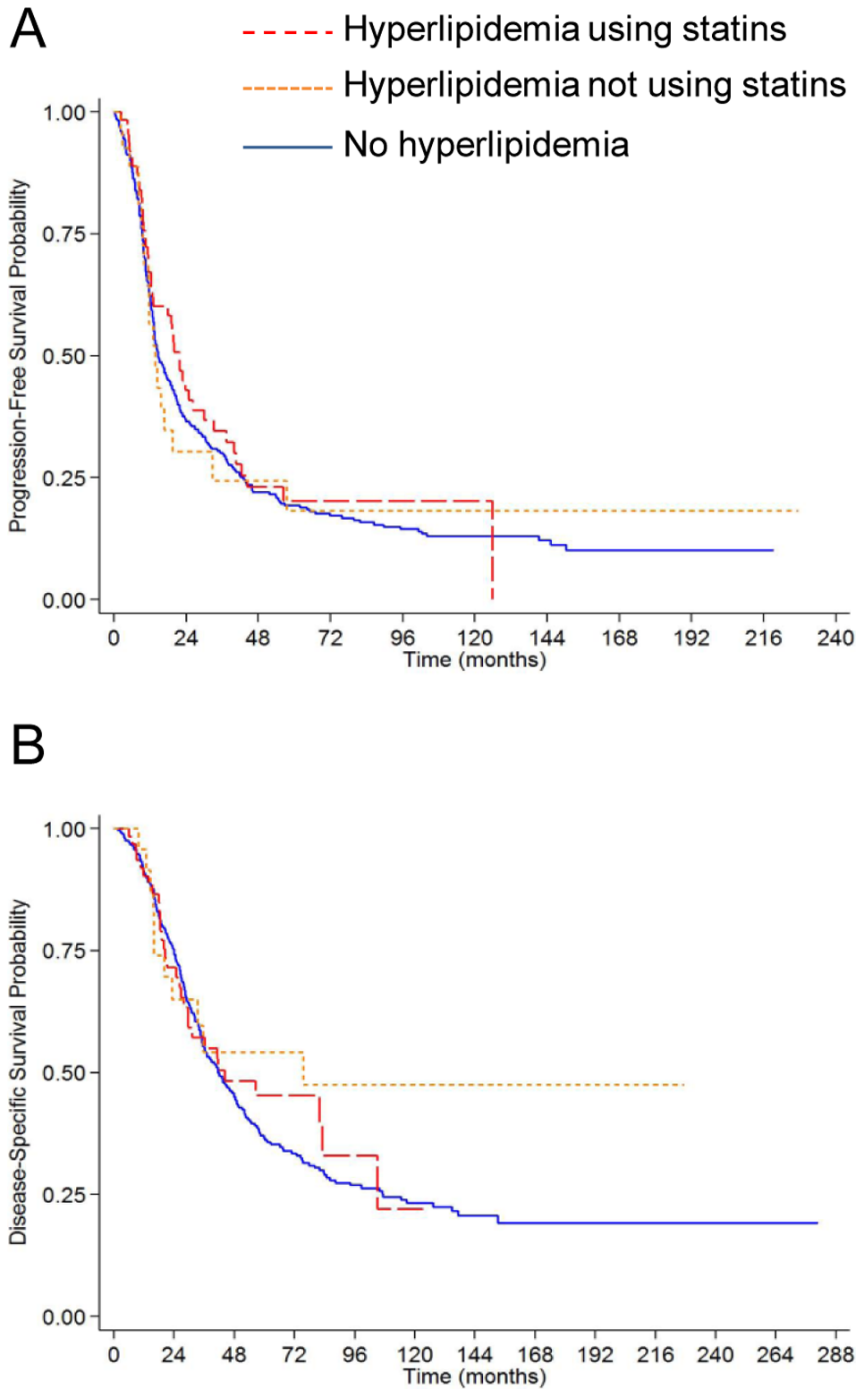
Kaplan-Meier estimates of survival outcomes in the entire cohort. The three groups are ovarian cancer patients with hyperlipidemia using statins (red long dashed line, n = 68), with hyperlipidemia not using statins (orange short dashed line, n = 28), and patients without hyperlipidemia (blue solid line, n = 346). *p* values are from the log-rank test. (**A**) Progression-free survival; (**B**) Disease-specific survival.

PFS was similar among the groups. Median PFS was 21.7 months (95% CI: 12.8–29.9 months) for hyperlipidemics using statins, 13.6 months (95% CI: 9.9–19.5) for hyperlipidemics not using statins, and 14.7 months (95% CI: 13.2–18.6 months) for non-hyperlipidemics (log-rank test comparing the three groups *p* = 0.69; Fig 1A). For DSS, we found that those patients with hyperlipidemia not using statins had a longer OvCa survival, but this was not statistically significant. The median DSS was 44.2 months (95% CI: 27.8–104.9 months) for patients with hyperlipidemia using statins, 75.7 months (95% CI: 20.2-non estimable) for patients with hyperlipidemia not using statins, and 41.5 months (95% CI: 35.3–48.5 months) for patients without hyperlipidemia (log-rank test comparing the three groups *p* = 0.43; Fig 1B). To adjust for potential confounders in the survival analysis, Cox proportional hazards models were fit. In the adjusted analysis, the hazards for disease recurrence (PFS) or disease-specific death (DSS) were not significantly lower in patients with hyperlipidemia taking statins when compared to patients without hyperlipidemia or to those hyperlipidemics who did not use statins (Table 3). In a two group direct comparison of hyperlipidemics using statins vs. hyperlipidemics not using statins, the hazards for PFS and DSS were similar (Table 3).

**Table 3:** Results of Cox Proportional Hazards Regression for Survival.

	Progression-Free Survival	P value	Disease-Specific Survival	P value
**Entire Cohort**				
No hyperlipidemia	1		1	
Hyperlipidemia not using statins	0.77 (0.44–1.35)	0.37	0.54 (0.28–1.04)	0.07
Hyperlipidemia using statins	0.84 (0.56–1.27)	0.41	0.80 (0.50–1.29)	0.37
**Hyperlipidemia only**				
Hyperlipidemia not using statins	1		1	
Hyperlipidemia using statins	1.09 (0.56–2.12)	0.80	1.48 (0.68–3.22)	0.32

Data are hazard ratio (95% confidence interval).

Adjusted for age at diagnosis, race, BMI, ASA class (PFS only), metformin use, residual tumor >1 cm, primary cytoreductive surgery, histologic subtype, FIGO stage, tumor site and grade.

**Figure 2:**
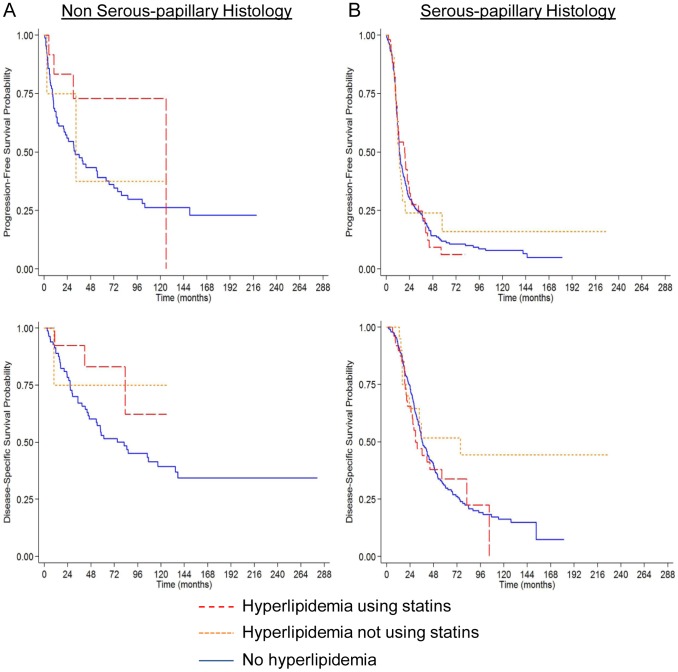
Kaplan-Meier estimates of survival outcomes by histologic subtype. The three groups are ovarian cancer patients with hyperlipidemia using statins (red long dashed line), with hyperlipidemia not using statins (orange short dashed line), and patients without hyperlipidemia (blue solid line). *P* values are from the log-rank test. (**A**) Subjects with non-serous-papillary OvCa. Progression-free and disease-specific survival. (**B**) Subjects with serous-papillary OvCa. Progression-free and disease-specific survival.

Clinically, OvCa is a heterogeneous disease with four different histologic subtypes, each with a unique clinical, genetic and molecular profile [45], [46]. Based on this, we hypothesized that response to statin use may vary by histologic subtype. Therefore, the cohort was divided into two groups: papillary serous (n = 334, 76%) and non-papillary serous (n = 106, 24%). A trend toward increased PFS and DSS was noted among statin users with non-papillary serous histologic subtypes (Fig 2A), but not among those with papillary serous histologic subtypes (Fig 2B). After controlling for confounders using Cox proportional hazards models, statin users with non-papillary serous OvCa, versus non-hyperlipidemics, had significantly improved PFS (HR = 0.23, 95% CI: 0.07–0.79, *p* = 0.02) and DSS (HR = 0.23, 95% CI: 0.05–0.96, *p* = 0.04) (Table 4; histologic subtype by hyperlipidemia cohort interaction *p* = 0.06 for PFS and *p* = 0.09 for DSS). To assure that changes in ovarian cancer treatment or statin use over time were not influencing the findings, two additional analyses were performed. First, the cohort was limited to a timeframe when statin use was more prevalent, patients treated from 2000–2013. Here the findings were the same as those from the analysis using the entire cohort. Specifically, improved PFS and DSS were noted among statin users with non-papillary serous histologic subtypes, but not among those with papillary serous histologic subtypes. In a second analysis, we tested whether there was a difference in DSS or PFS based on treatment year and found no association (*p*>0.80 for both DSS and PFS).

**Table 4:** Results of Cox Proportional Hazards Regression for Survival by Histologic Subtype.

	Progression-Free Survival	P value	Disease-Specific Survival	P value
**Non-serous papillary**				
No hyperlipidemia	1		1	
Hyperlipidemia not using statins	0.85 (0.11–6.45)	0.88	1.45 (0.19–11.09)	0.72
Hyperlipidemia using statins	0.23 (0.07-0.79)	0.02	0.23 (0.05–0.96)	0.04
**Serous papillary**				
No hyperlipidemia	1		1	
Hyperlipidemia not using statins	0.78 (0.43–1.39)	0.40	0.52 (0.26–1.03)	0.06
Hyperlipidemia using statins	1.06 (0.69–1.64)	0.78	1.01 (0.62–1.64)	0.98

Data are hazard ratio (95% confidence interval).

Adjusted for age at diagnosis, race, BMI, ASA class (PFS only), metformin use, residual tumor >1 cm, primary cytoreductive surgery, FIGO stage, tumor site and grade.

**Figure 3:**
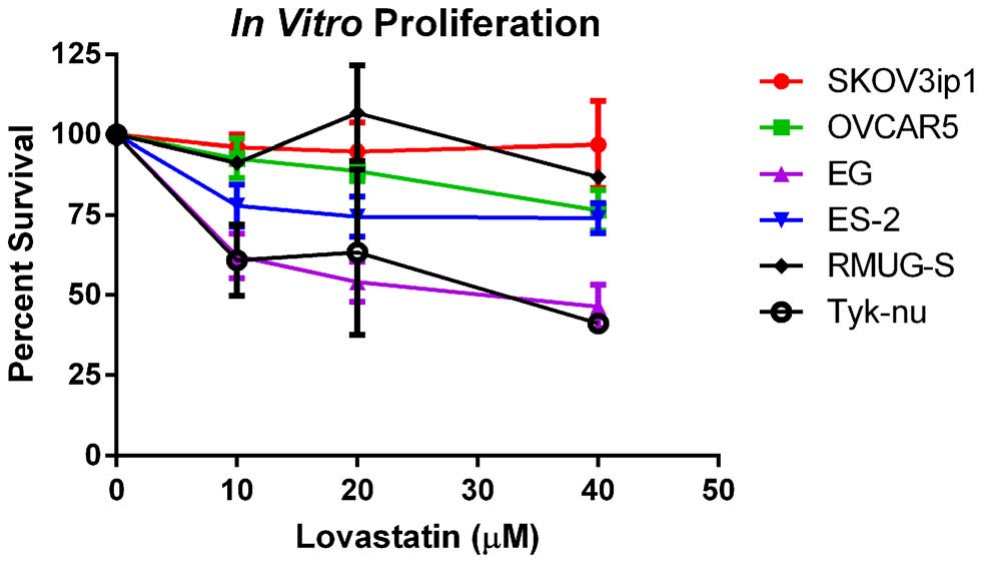
*In vitro* proliferation assays. We used different OvCa cell lines which correspond to various histological subtypes of OvCa: SKOV3ip1 (adeno-carcinoma), OVCAR5 (adeno-carcinoma), EG (endometrioid), ES-2 (clear cell), RMUG-S (mucinous) and TYK-nu (serous-papillary). Cells were plated in quintuplicate into 96-well plates and treated with varying doses of lovastatin (10 µM, 20 µM and 40 µM) for 24 hours. The MTT proliferation assay was then performed. Experiments were performed three times. Y-axis represents percent of living cells compared to untreated control group. X-axis represents dose of lovastatin in µM.

To explore, *in vitro*, the finding of a differential effect of statins based on histologic subtype cell lines of various histologic subtypes were treated with lovastatin and cellular proliferation was measured. Lovastatin had a variable effect in the different cell lines. In two cell lines (SKOV3ip1 and RMUG-S), lovastatin inhibited growth, and in the remaining cell lines the drug induced cell death (Fig 3). However, *in vitro*, the papillary serous cell line (TYK-nu) showed the greatest growth inhibition with statin treatment, a result inconsistent with the findings from the patient data set.

For a more detailed interrogation of statins' effect in OvCa, two additional areas were investigated. First, since the primary tumor site was different in the three groups (Table 2), we asked if statin use was more likely to be associated with improved survival in patients with a particular tumor site. We found that, in an analysis stratified by tumor site (ovary vs. non-ovary), statin use was not associated with improved OvCa survival in either tumor site group. In a second area of investigation, we evaluated statin formulation. Preclinical studies indicate that lipophilic (simvastatin, atorvastatin, lovastatin, fluvastatin, pitavastatin), but not hydrophilic statins (rosuvastatin and pravastatin), have anti-cancer properties [47]. Therefore, we repeated the analysis comparing patients using lipophilic statins (n = 59) to patients using hydrophilic statins (n = 37) and patients without hyperlipidemia (n = 346). Contrary to the findings published in pre-clinical studies, lipophilic statin use was not associated with improved PFS or DSS. The median PFS for patients using a lipophilic statin was 21.8 months (95% CI: 13.0–33.0) compared to 14.6 months (95% CI: 13.2–17.5) for non-users of lipophilic statins (*p* = 0.42). The median DSS was 41.3 months (95% CI: 27.8–83.1) for lipophilic statin users and 41.9 (95% CI: 35.4–48.9) months for non-users (*p* = 0.69). Multivariate analysis also did not demonstrate significantly improved PFS (HR = 0.94; 95% CI: 0.62–1.44; *p* = 0.78) or DSS (HR = 0.88, 95% CI: 0.54–1.43; *p* = 0.60) among users of lipophilic statins.

## Discussion

There is increasing epidemiologic and laboratory based evidence suggesting that statins, a class of medications widely used to treat elevated cholesterol, may have anti-cancer effects. In this study, we show that, in a single institution retrospective cohort, statin use is associated with improved OvCa survival with hazard ratios equal to 0.23 for both progression-free survival and disease specific survival (PFS, *p* = 0.02; DSS, *p* = 0.04). Of note, the protective effect of statins was limited to patients with non-serous-papillary subtypes of OvCa (Table 4).

This study adds to our knowledge of statin use and OvCa in two new and important ways. First, our analysis was OvCa histologic subtype-specific and indicates that the protective effect of statins may be limited to patients with non-serous-papillary subtypes of OvCa. Second, the study took into account the possibility that the effect of statins in cancer is primarily attributable to the presence of hyperlipidemia [48], [49] by dividing the cohort into three groups: patients without hyperlipidemia, patients with hyperlipidemia not using statins, and patients with hyperlipidemia using statins. Prior publications have only compared statin users to non-users [25], [50].

Overall, the findings reported here indicating a protective effect of statins in OvCa add to those from Elmore *et. al.* in 2008 [25] and a more recent report of an association between statin use and improved outcomes in multiple gynecologic malignancies, including ovarian [50]. However, one apparent discrepancy exists between our findings and those of Elmore *et. al*. [25]. We found no significant association between statin use and improved survival among patients with papillary serous subtype of OvCa. In contrast, 90% of the cohort in the Elmore *et. al*. study had papillary serous subtype and statin use was associated with improved survival. These incongruent findings may be due to the fact that our cohort had a higher rate of stage IV disease (22% vs. 12%) and a higher percent of patients with >1 cm of residual disease after cytoreductive surgery (31% vs. 17%). Perhaps, in the setting of the highly aggressive serous-papillary histologic subtype, advanced stage, and residual disease >1 cm, the protective effect of statins was lost. In the analysis reported here, it would have been informative to know the specific histologic subtypes for which statins were most protective. Unfortunately, due to sample size limitations we were not able to perform survival analysis for the individual histologic subtypes and had to limit our analysis to papillary serous versus non-papillary serous cancers.

While there is increasing epidemiologic evidence of a protective effect of statins in cancer in general [6]–[8], and this report adds to the findings in OvCa, the anti-cancer mechanism(s) of action of statins remain largely unknown. At least two possibilities exist. The first, and most well studied possibility, is that statins protect against OvCa by decreasing isoprenoid production and subsequently inhibiting cancer signaling pathways [21]–[23]. The second possibility is that statins may protect against OvCa by reducing serum LDL. This is biologically plausible since it has been reported that patients with elevated LDL have worse OvCa survival [49]. The molecular mechanism of action of statins in OvCa is an ongoing area of investigation in our laboratory and others.

In conclusion, tapping into the anti-cancer effects of drugs used for non-cancer indications could represent a provocative new approach to cancer drug development. A precedent for this paradigm has already been set with the diabetic medication, metformin. Currently, the body of literature supporting anti-cancer effects of metformin is larger than those supporting anti-cancer effects of statins. Numerous studies have indicated that metformin has an anti-cancer effect in several cancers, including ovarian [33], [34], and, as of April 2014, the diabetes drug is being tested prospectively in 17 randomized clinical trials as a cancer treatment in patients without diabetes [51]. Whether a similar trajectory can be set for statins remains to be determined. Our results, showing improved OvCa survival among statin users, support a growing body of evidence of an anti-cancer effect of statins. However, larger epidemiologic studies and further pre-clinical testing are required to identify the molecular mechanisms of action mediating the anti-cancer effects of statins before a prospective study can be contemplated.
